# The Extended Transmembrane Orai1 N-terminal (ETON) Region Combines Binding Interface and Gate for Orai1 Activation by STIM1*[Fn FN3]^♦^

**DOI:** 10.1074/jbc.M113.501510

**Published:** 2013-08-13

**Authors:** Isabella Derler, Peter Plenk, Marc Fahrner, Martin Muik, Isaac Jardin, Rainer Schindl, Hermann J. Gruber, Klaus Groschner, Christoph Romanin

**Affiliations:** From the ‡ Institute of Biophysics, Johannes Kepler University of Linz, Gruberstrasse 40, 4020 Linz and; the § Institute of Biophysics, Medical University of Graz, Harrachgasse 21/4, 8010 Graz, Austria

**Keywords:** Calcium Channels, Calcium Intracellular Release, Calcium Signaling, Electrophysiology, Ion Channels

## Abstract

STIM1 and Orai1 represent the two molecular key components of the Ca^2+^ release-activated Ca^2+^ channels. Their activation involves STIM1 C terminus coupling to both the N terminus and the C terminus of Orai. Here we focused on the extended transmembrane Orai1 N-terminal (ETON, aa73–90) region, conserved among the Orai family forming an elongated helix of TM1 as recently shown by x-ray crystallography. To identify “hot spot” residues in the ETON binding interface for STIM1 interaction, numerous Orai1 constructs with N-terminal truncations or point mutations within the ETON region were generated. N-terminal truncations of the first four residues of the ETON region or beyond completely abolished STIM1-dependent Orai1 function. Loss of Orai1 function resulted from neither an impairment of plasma membrane targeting nor pore damage, but from a disruption of STIM1 interaction. In a complementary approach, we monitored STIM1-Orai interaction via Orai1 V102A by determining restored Ca^2+^ selectivity as a consequence of STIM1 coupling. Orai1 N-terminal truncations that led to a loss of function consistently failed to restore Ca^2+^ selectivity of Orai1 V102A in the presence of STIM1, demonstrating impairment of STIM1 binding. Hence, the major portion of the ETON region (aa76–90) is essential for STIM1 binding and Orai1 activation. Mutagenesis within the ETON region revealed several hydrophobic and basic hot spot residues that appear to control STIM1 coupling to Orai1 in a concerted manner. Moreover, we identified two basic residues, which protrude into the elongated pore to redound to Orai1 gating. We suggest that several hot spot residues in the ETON region contribute in aggregate to the binding of STIM1, which in turn is coupled to a conformational reorientation of the gate.

## Teaser

**Background:** STIM1 and Orai1, reconstituting a main cellular Ca^2+^ entry pathway, interact via their cytosolic strands.

**Conclusion:** Several “hot spot” residues in the ETON region mediate STIM1 interaction, enabling conformational reorientation of the gate.

**Significance:** Identification of critical residues for protein-protein interaction are fundamental to therapeutic drug development.

## Introduction

The CRAC^4^ channel signaling machinery consists of two key components, STIM1 (1, 2) and Orai (3–5). STIM1 acts as an endoplasmic reticulum-located Ca^2+^ sensor and mediates CRAC channel activation (1, 2, 6). After endoplasmic reticulum Ca^2+^ store depletion, STIM1 multimerizes and redistributes into punctae close to the plasma membrane (7, 8). It undergoes a switch to an extended conformation, thereby coupling to the CRAC channel Orai (9, 10). Finally, the coupling of STIM1 to Orai culminates in CRAC channel activation, which allows Ca^2+^ influx into the cell (11–14).

Recently, Hou *et al.* (15) have published the crystal structure of *Drosophila* Orai. It exhibits a hexameric assembly of Orai subunits with the ion pore located in the center, which is surrounded by the transmembrane domains. Thereby the first transmembrane domains form an inner ring around the ion pore, the second and the third ones form a middle ring, and the fourth transmembrane domains form the outer ring (15). Ca^2+^ enters the cell at a 6 Å narrow opening: the selectivity filter, which is composed of the glutamate Glu-106 in human Orai1 (16). Toward the cytoplasmic side, the pore opens to a wider cavity including hydrophobic side chains such as valine, phenylalanine, and lysine: for example, Val-102. The mutation of Val-102 to an alanine or a cysteine profoundly alters the selectivity of the pore and leads to constitutively active nonselective currents (17). Upon STIM1 binding, Orai1 V102A regains Ca^2+^ selectivity comparable with wild-type Orai1 (17). The selectivity filter and the hydrophobic cavity are followed by a flexible glycine hinge (Gly-98) (18), which may enable flexion of the upstream pore-lining region to reduce the impedance of Ca^2+^ flow after passing the selectivity filter (16).

Strikingly, this part of the cytosolic N-terminal strand upstream of the first transmembrane helix (TM1) forms a helical (19), extended transmembrane Orai1 N-terminal (ETON) region that comprises the N-terminal residues aa73–90, which are fully conserved among the three human homologues of Orai proteins and protrudes about 20 Å into the cytosol (16). The TM1 helix together with the ETON region contains three positively charged residues Arg-91, Lys-87, and Arg-83, which directly line the pore and thus have been supposed to form an electrostatic barrier impeding Ca^2+^ flow when the channel is in the closed state (16). The arginine Arg-91 inhibits store-operated current activation upon its mutation to a hydrophobic residue (20, 21). This barrier of the three positively charged residues must be released to let Ca^2+^ pass into the cell, which may be accomplished by an interaction of STIM1 with the conserved ETON regions forming the elongated pore (16). The CRAC-activating domain (CAD), a small Orai-activating STIM1 C-terminal fragment, has already been shown to interact with an N-terminal fragment (73–90) of Orai1 (22), underlining its relevance as the second major interaction site besides Orai1 C terminus (11, 12, 23). Orai1 is probably gated by a STIM1 binding to bridge the cytosolic TM1 and TM4 extended helices, thereby applying a force at the helical TM1 extension to form and stabilize the open pore state (16).

Another positively charged residue near the membrane, Lys-85 (24, 25), located on the pore-averted side of the helical TM1 extension, has been reported to abolish store-operated activation upon a K85E mutation due to a defect in gating together with a weaker STIM1 binding (24, 25).

In this study, we performed a systematic screen along the conserved ETON region to determine potential “hot spot” (26–28) residues in the binding interface with STIM1. A combined approach based on Orai1 N-terminal truncations and point mutations revealed that almost the whole ETON region provides an elongated low affinity binding interface for the interaction with STIM1. It additionally contains electrostatic gating elements that fine-tune and shape the elongated pore for controlled STIM1-dependent Ca^2+^ entry.

## EXPERIMENTAL PROCEDURES

### Molecular Biology

For N-terminal fluorescence labeling of human Orai1 (Orai1; accession number NM_032790, provided by the A. Rao laboratory), the constructs were cloned into the pEYFP-C1 (Clontech) expression vector via KpnI and XbaI restriction sites. For the alanine scanning mutagenesis, pEYFP-C1-Orai1 served as template. Site-directed mutagenesis (pEYFP-Orai1 S75W, S75A/W76A, W76A, W76P, W76S, W76E, W76R, L74S/W76S, L74R/W76R, L74E/W76E, R77A/K78A, R83A/K87A, K85E, R83E/K87E, N_1–74_ S75A/W76A, ΔN_1–74_ R83A/K87A, ΔN_1–76_ R83A/K87A) was performed using the QuikChange^TM^ XL site-directed mutagenesis kit (Stratagene). pEYFP-Orai1 N-terminal deletion mutants (Orai1 ΔN_1–38_, ΔN_1–47_, ΔN_1–72_, ΔN_1–74_, ΔN_1–75_, ΔN_1–76_, ΔN_1–77_, ΔN_1–78_, ΔN_1–80_, ΔN_1–82_, ΔN_1–85_, ΔN_1–87_, DN_1–88_, ΔN_1–89_) were amplified via PCR including an N-terminal KpnI and a C-terminal XbaI restriction site for cloning into the pEYFP-C1 vector. V102A mutants were accomplished analogously (using the QuikChange^TM^ XL site-directed mutagenesis kit (Stratagene) with the corresponding point mutants serving as template.

Human STIM1 (STIM1; accession number: NM_003156, provided by the T. Meyers laboratory) was subcloned from pcDNA3.1/V5-His TOPO via KpnI and ApaI restriction sites into a custom made mCherry vector (peGFPN-3 as backbone). pECFP-C1 STIM1 C terminus (aa233–685 WT and L251S) was used as template for the generation of pECFP-OASF (WT and L251S) by introducing a stop codon at position 475 (aa233–474) using the QuikChange XL site-directed mutagenesis kit (Stratagene). pEYFP-C1 STIM1 C terminus (aa233–685 WT and L251S) was used as template for the generation of pEYFP-OASF (WT and L251S) by introducing a stop codon at position 475. pEYFP-C1 STIM1 C terminus (aa233–685 WT) was used as template for the generation of pEYFP-233–485 by introducing a stop codon at position 486. All point mutations were performed using the QuikChange XL site-directed mutagenesis kit (Stratagene). STIM1 fragment 344–449 was amplified via PCR including an N-terminal KpnI and a C-terminal XbaI restriction site for cloning into the pECFP-C1 vector. All clones were confirmed by sequence analysis.

### Biotinylation

#### 

##### Reagents

EZ-Link Sulfo-NHS-LC-biotin and high capacity streptavidin-Agarose resin were purchased from Pearce; anti-Orai1 and anti-β actin antibodies were from Sigma-Aldrich; anti-rabbit IgG peroxidase-linked was from Amersham Biosciences.

##### Biotinylation of Cell Surface Membrane Proteins

Cell biotinylation was performed as described previously (29). Briefly, HEK293 cells were transfected with YFP-Orai1 WT or different YFP-Orai1 mutants. After 24–72 h, cell surface proteins were incubated for 1h at 4 °C using EZ-Link sulfo-NHS-LC-biotin (0.5 mg/ml; Pierce). After incubation, 100 mm Tris was added to stop the reaction. Cells were washed twice with PBS to remove excess biotinylation agent and lysed with lysis buffer, pH 8.0, containing 100 mm NaCl, 20 mm Tris, 2 mm EDTA, 10% glycerol, 0.5% Nonidet P40 and supplemented by 20 μl/ml protease inhibitor mixture (Roche Applied Science). Lysed samples were centrifuged at 14,000 × *g* for 15 min. Finally, biotinylated proteins in the supernatant were precipitated using high capacity streptavidin-Agarose resin (Pearce) overnight at 4 °C on a rocking platform. The samples were resolved by 12% SDS-PAGE, and protein detection was achieved using the anti-Orai1 antibody diluted 1:2000 in PBS-Tween-20 (PBST). The primary antibody was removed, and blots were washed six times for 5 min each with PBST. To detect the primary antibody, blots were incubated for 1 h with horseradish peroxidase-conjugated rabbit anti-mouse IgG antibody diluted 1:5000 in PBST and then with enhanced chemiluminescence reagents for 2 min. Blots were then exposed to photographic films. The density of bands on the film was measured using scanning densitometry. Anti-β-actin (A5441; Sigma) was used to normalize protein expression.

### Electrophysiology

Electrophysiological recordings comparing characteristics of 2–3 constructs were carried out in paired comparison on the same day. Expression patterns and levels of the various constructs were carefully monitored by confocal fluorescence microscopy and were not significantly changed by the introduced mutations. Electrophysiological experiments were performed at 20–24 °C, using the patch clamp technique in the whole-cell recording configuration. For STIM1/Orai as well as STIM1 C terminus/Orai current measurements, voltage ramps were usually applied every 5 s from a holding potential of 0 mV, covering a range of −90 to +90 mV over 1 s. The internal pipette solution for passive store depletion contained (in mm) 3.5 MgCl_2_, 145 cesium methanesulfonate, 8 NaCl, 10 HEPES, 20 EGTA, pH 7.2. Extracellular solution consisted of (in mm) 145 NaCl, 5 CsCl, 1 MgCl_2_, 10 HEPES, 10 glucose, 10 CaCl_2_, pH 7.4.

### Confocal Fluorescence Microscopy

Confocal microscopy for co-localization experiments was performed similarly as in Ref. 30. In brief, a QLC100 real-time confocal system (VisiTech International) was used for recording fluorescence images connected to two Photometrics CoolSNAPHQ monochrome cameras (Roper Scientific) and a dual port adapter (dichroic, 505lp; cyan emission filter, 485/30; yellow emission filter, 535/50; Chroma Technology Corp.). This system was attached to an Axiovert 200M microscope (Zeiss) in conjunction with an argon ion multiwavelength (457, 488, 514 nm) laser (Spectra Physics). The wavelengths were selected by an acousto-optical tunable filter (VisiTech International). MetaMorph 5.0 software (Universal Imaging Corp.) was used to acquire images and to control the confocal system. Illumination times for CFP and YFP images that were recorded with a minimum delay consecutively were about 900 ms.

### Thermophoresis Measurements

The thermophoresis experiments were carried out with the monolith NT.115 (Nanotemper). We employed the STIM1 233–485 C-terminal fragment as a surrogate for full-length STIM1 as it expressed much better in HEK293 cells than the shorter STIM1-OASF. In brief, YFP-STIM1 233–485 transfected HEK293 cells were washed 24 h after transfection with PBS and pelleted at 1500 × *g* for 10 min. The supernatant was discarded, and the pellet was resuspended in 350 μl of DMEM. The cells were lysed by adding 35 μl of lysis buffer (20 mm TRIS (pH 7.4), 1% Nonidet P40, 150 mm NaCl, and protease inhibitor) for 30 min and afterward centrifuged at 13,000 × *g* for 20 min. The supernatant containing YFP-STIM1 233–485 was stored at 4 °C until analysis. Orai1 peptides (aa70–91, aa77–91, aa70–91 L74E/W76E) were purchased from peptide 2.0. Up to 16 samples of different dilutions were prepared (concentration of YFP-STIM1 233–485 was kept constant, concentration of the Orai1 peptide was varied), and after an incubation time of 5 min, the reaction was transferred into glass capillaries and placed on the sample tray. The instrument recognized automatically the capillaries, and upon measuring the thermophoresis signal, the dissociation coefficient was calculated.

## RESULTS

### 

#### 

##### STIM1-dependent Activation of Orai1 Requires Almost the Whole ETON Region

**FIGURE 1.**
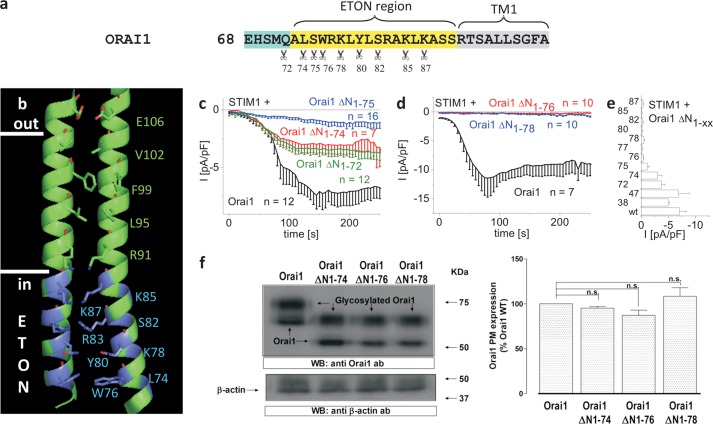
**N-terminal truncations including Trp-76 of the ETON region or beyond abolish STIM1-dependent Orai1 function.** *a*, amino acid sequence of Orai1 N terminus displaying those residues (indicated by *scissors*) in the conserved ETON region (*yellow area*) up to which increasing N-truncations have been performed. *b*, schematic representation of two TM1 domains + ETON regions highlighting those residues up to which N-truncations have been performed (*blue*) in the ETON region. *c*, time course of whole cell inward currents at −74 mV activated by passive store depletion of HEK 293 cells co-expressing CFP-STIM1 and YFP-Orai1 ΔN_1–72_, ΔN_1–74_ and ΔN_1–75_ in comparison with wild-type YFP-Orai1. *pF*, picofarads. *d*, time course of whole cell inward currents at −74 mV activated by passive store depletion of HEK293 cells co-expressing CFP-STIM1 and YFP-Orai1 ΔN_1–76_ and ΔN_1–78_ in comparison with wild-type YFP-Orai1. *e*, block diagram comparing maximal current densities of Orai1 N-truncation mutants (ΔN_1–38_, _-47_, _-72_, _-74_, _-75_, _-76_, _-77_, _-78_, _-80_, _-82_, _-85_, _-87_) with that of wild-type Orai1. *Error bars* indicate S.E. *f*, Orai1 plasma membrane expression in HEK293 cells expressing Orai1 WT, Orai1 ΔN_1–74_, Orai1 ΔN_1–76_, or Orai1 ΔN_1–78_ determined by biotinylation as described under “Experimental Procedures” (*n* = 5; *, *p* < 0.05). *Error bars* indicate S.E. *n.s.*, not significant. *WB*, Western blot.; *ab*, antibody.

The ETON region (aa73–90, Fig. 1, *a* and *b*), comprising the extended TM1 Orai1 N-terminal helix, is essential for store-operated CRAC current activation via STIM1 (11, 12, 22–24). For a thorough evaluation of the impact of this ETON region on STIM1 binding and Orai1 activation, we initially generated several Orai1 mutants with increasing N-terminal deletions in an attempt to uncover the minimal stretch of the ETON region that retains store-operated activation. The function of these Orai1 truncation mutants co-expressed in HEK293 cells with STIM1 was characterized by employing the whole-cell patch clamp technique. Orai1 ΔN_1–38_ partially and Orai1 ΔN_1–47_ fully lacking the arginine-/proline-rich region were activated via STIM1 in a comparable manner as wild-type Orai1 (supplemental Fig. 1*a*). Orai1 ΔN_1–72_ and Orai1 ΔN_1–74_ still displayed STIM1-dependent current activation, which was similar to that of the Orai1 ΔN_1–73_ mutant (31), although slightly reduced when compared with Orai1 wild-type (Fig. 1, *c* and *e*). Deletion of a further residue leading to Orai1 ΔN_1–75_ retained store-operated activation, yet further reduced current density (Fig. 1, *c* and *e*). However, the following Trp-76 (Orai1 ΔN_1–76_) or consecutive residues (Arg-77/Lys-78/Y80/Ser-82/Lys-85/Lys-87), when deleted, completely abolished STIM1-dependent CRAC current activation (Fig. 1, *d* and *e*). In line with this finding, STIM1 C-terminal fragments such as STIM1 344–449 or OASF (STIM1 233–474), both displaying an increased coupling to Orai1 proteins, thereby enhancing currents 2–3-fold (32), also failed to activate Orai1 ΔN_1–76_ and Orai1 ΔN_1–78_ (supplemental Fig. 1, *b* and *c*).

To exclude impaired plasma membrane targeting of the respective Orai1 deletion mutants as the cause of loss of function, we estimated the amount targeted to the plasma membrane by biotinylation experiments. These studies revealed similar plasma membrane expression levels for YFP-labeled Orai1, Orai1 ΔN_1–76_, and Orai1 ΔN_1–78_ (Fig. 1*f*) in line with similar plasma membrane fluorescence intensities (data not shown).

Hence, based on these N-terminal deletion experiments, most (aa76–90) of the ETON region (aa73–90) is essential for STIM1-dependent Orai1 activation. As the loss of function is not caused by impairment of plasma membrane expression, these truncations apparently affected either STIM1 coupling or Orai1 gating.

##### Orai1 V102A Currents Enable Detection of STIM1 Interaction by a Rightward Shift in the Reversal Potential

In a complementary approach, we utilized the recently reported constitutively active Orai1 mutant V102A/V102C (17) to examine the impact of the N-terminal deletions on channel function and coupling to STIM1. Specifically, the V102A/V102C mutant is able to gate in the absence of STIM1, yielding constitutively active but nonselective currents, which regain selectivity upon coupling to STIM1 (supplemental Fig. 2, *a–e*) (17). The Orai1 mutant V102A/L273S or V102C/L276D (17) with a disrupted C-terminal binding site for STIM1 failed to re-establish Ca^2+^ selectivity as judged from a lack of rightward shift in the reversal potential in the presence of STIM1 (supplemental Fig. 2, *c–e*) in line with the predominant role of Orai1 C terminus in the coupling to STIM1. As interactions of both the C termini and the N termini of Orai1 with STIM1 are required for a functional channel (12, 22, 24, 33), we hypothesize that the shift in the reversal potential is induced by STIM1 binding to Orai1 N-terminal regions, as long as the Orai1 C terminus is intact. Thus, we utilized the potential rightward shift in the reversal potential as a read-out parameter for an N-terminal interaction of Orai1 with STIM1.

N-terminal deletion mutants up to residues 38, 72, and 74, which retained STIM1-dependent Orai1 currents when introduced into the Orai1 V102A core, yielded constitutively active currents that were nonselective in the absence of STIM1 and Ca^2+^-selective in its presence. This was clearly evident from the rightward shift in the reversal potential similar to wild-type Orai1 (supplemental Fig. 2, *f–j*). Thus, the N-terminal strand of Orai1 including residue 74 was dispensable for STIM1-mediated high Ca^2+^ selectivity of Orai1 in line with the intact ETON region coupling to STIM1.

**FIGURE 2.**
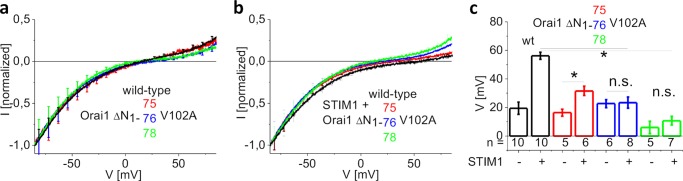
**Nonfunctional Orai1 N-truncation mutants introduced into the Orai1 V102A core display no shift in reversal potential in the presence of STIM1.** *a*, I/V relationships of normalized Orai1 V102A currents in comparison with those of Orai1 ΔN_1–75_ V102A, Orai1 ΔN_1–76_ V102A, and Orai1 ΔN_1–78_ V102A. *b*, analogue to *panel a* in the presence of STIM1. *c*, block diagram displaying reversal potentials of Orai1 V102A, Orai1 ΔN_1–75_ V102A, Orai1 ΔN_1–76_ V102A, and Orai1 ΔN_1–78_ V102A currents in the absence and presence of STIM1 (*, *p* < 0.05). *Error bars* indicate S.E. *n.s.*, not significant.

When introduced into the Orai1 V102A core, the substantially less active Orai1 ΔN_1–75_ as well as the other nonfunctional N-truncation mutants Orai1 ΔN_1–76_ and ΔN_1–78_ (Fig. 1, *c* and *d*) displayed constitutive, nonselective currents with a reversal potential between +10 mV and +20 mV (Fig. 2, *a* and *c*), suggesting that these N-truncations did not severely damage the pore. This was also in line with the preserved 2-aminoethoxydiphenyl borate-evoked currents obtained with the nonfunctional truncation mutants (supplemental Fig. 3, *a–d*). Although the Orai1 ΔN_1–75_ V102A channels when co-expressed with STIM1 displayed a significant rightward shift in the reversal potential of constitutive currents, the previously (see Fig. 1) STIM1 nonresponsive Orai1 N-truncation mutants ΔN_1–76_ to ΔN_1–82_ with the V102A mutation lacked this shift in the reversal potential when co-expressed with STIM1 (Fig. 2, *b* and c and supplemental Fig. 4). Deletion of further residues up to aa85 resulted in smaller but still constitutively active, nonselective currents with a reversal potential around 0 mV independent of the presence or absence of STIM1 (supplemental Fig. 4). Truncation up to aa87 or of the whole NT resulted in almost nonfunctional channels (supplemental Fig. 4).

In summary, these experiments established the inducible Ca^2+^ selectivity for Orai1 V102A N-terminal deletion mutants as a read-out parameter for detection of STIM1 interaction. The results obtained were perfectly in line with the previous experiments shown in Fig. 1 in that residues around aa76 of the ETON region were essential in contributing to the Orai1 N-terminal interaction with STIM1.

##### STIM1 Interaction with the Nonfunctional Orai1 N-terminal Deletion Mutants Is Significantly Reduced

**FIGURE 3.**
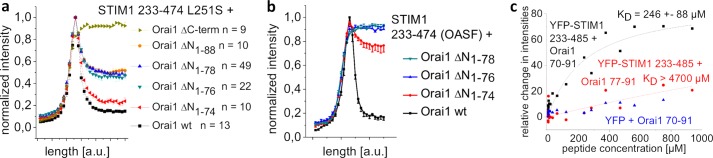
**Nonfunctional Orai1 N-truncation mutants display a significant reduction in STIM1 binding.** *a*, intensity plots representing the localization of STIM1 233–474 L251S across the cell when co-expressed with Orai1 ΔN_1–74,_ Orai1 ΔN_1–76_, Orai1 ΔN_1–78_, Orai1 ΔN_1–88_, and Orai1 ΔC-term when compared with wild-type Orai1. *a. u.*, arbitrary units. *b*, intensity plots representing the localization of STIM1 233–474 (OASF) across the cell when co-expressed with Orai1 ΔN_1–74_, Orai1 ΔN_1–76_, and Orai1 ΔN_1–78_ when compared with wild-type Orai1. *c*, binding curve fitted to a one-site binding model for YFP-STIM1 233–485 from a HEK293 cell lysate together with Orai1 NT(aa70–91) or Orai1 NT(aa77–91) in comparison with YFP from HEK293 cell lysate together with Orai1 NT(aa70–91), obtained from thermophoresis experiments.

In a further approach, we employed co-localization experiments to directly visualize a potentially reduced coupling of the nonfunctional truncation mutants Orai1 ΔN_1–76_ and Orai1 ΔN_1–78_ with the co-expressed STIM1 OASF (aa234–474) as well as the OASF L251S fragment (Fig. 3, *a* and *b*). Initially, we employed OASF L251S, which already provides the active, extended conformation (9, 10). Co-expression of OASF L251S with wild-type Orai1 resulted in almost 90% co-localization, leaving 10% OASF L251S in the cytosol as judged from cell density profiles (Fig. 3*a*). The cytosolic fraction was increased to ∼20% when co-expressed with Orai1 ΔN_1–74_, compatible with the reduced current density in comparison with wild-type Orai1. The nonfunctional Orai1 ΔN_1–76_ and the Orai1 ΔN_1–78_ displayed ∼50% attenuation in co-localization with OASF L251S, similar to Orai1 ΔN_1–88_ where almost the whole N terminus was deleted. An Orai1 mutant lacking the whole C terminus (Orai1 ΔC-term) exhibited a complete loss of co-localization with OASF L251S as evident from the density profile and confirmed the dominant role of Orai1 C terminus in the coupling process (12). Next, we utilized the wild-type OASF, which requires interaction with Orai1 to adopt the extended conformation (9). The coupling of OASF to Orai1 ΔN_1–74_ was dramatically reduced with about 80% determined in the cytosol, whereas Orai1 ΔN_1–76_ and Orai1 ΔN_1–78_ completely lacked co-localization (Fig. 3*b*) similar to Orai1 ΔN_1–88_ with almost the whole N terminus missing (data not shown). Hence, OASF, when compared with OASF L251S, was particularly sensitive to Orai1 N-terminal deletions in that it apparently required interaction with both the N-terminal strands as well as the C-terminal strands of Orai1. Therefore, the OASF fragment represented another sensitive tool for detection of hot spot residues (see next paragraph) in the ETON region binding interface with STIM1.

For a more quantitative analysis, we utilized thermophoresis measurements to estimate the dissociation constant *K_D_* of the slightly extended OASF fragment YFP-STIM1 233–485 and the Orai1 N-terminal peptide aa70–91 containing the whole ETON region or the peptide aa77–91 corresponding to Orai1 ΔN_1–76_, which previously displayed a reduced interaction with STIM1. In accordance to both the functional (Figs. 1 and 2) and the co-localization (Fig. 3, *a* and *b*) experiments, we estimated (Fig. 3*c*) a rather weak *K_D_* of 246 ± 88 μm for the interaction between STIM1 233–485 and the ETON region (Orai1 aa70–91), whereas the truncated ETON segment (Orai1 aa77–91) exhibited no saturating binding curve (*K_D_*>4700 μm). In this behavior, the latter resembled that of free YFP with Orai1 aa70–91, corresponding to the negative control (Fig. 3*c*).

In summary, complete loss of STIM1-dependent Orai1 activation with progressive N-terminal deletions, including residue 76 or beyond, arises from a lack or an improper STIM1 binding to the truncated ETON region. In the following, we focused on characterizing hot spot residues in the ETON region that constitutes the binding interface with STIM1 for Orai1 current activation.

##### The Hydrophobic Residues Leu-74 and Trp-76 in the ETON Region Contribute in a Concerted Manner to Orai1-STIM1 Coupling

The serial N-terminal deletions examined above already revealed that amino acids downstream to Trp-76 were essential for STIM1 binding to Orai1 N terminus. Hence, we initially focused on that residue as a potential hot spot (34) within the binding interface of the ETON region, essential for STIM1 binding to and STIM1-mediated gating of Orai1 channels. However, several single point mutations of Trp-76 tested (W76A/W76S/W76R/W76E; supplemental Fig. 5*a*), except W76P, did not exert a substantial effect on STIM1-dependent current activation as drastic as was found with the nonfunctional Orai1 ΔN_1–76_. The loss of function upon proline substitution in the N terminus of Orai1 W76P, which dramatically interfered with its function (supplemental Fig. 5, *b* and *c*), probably by generating a kink in the ETON region, might imply that indeed the region around Trp-76 but not the residue alone was indispensable for STIM1-dependent Orai1 activation.

**FIGURE 4.**
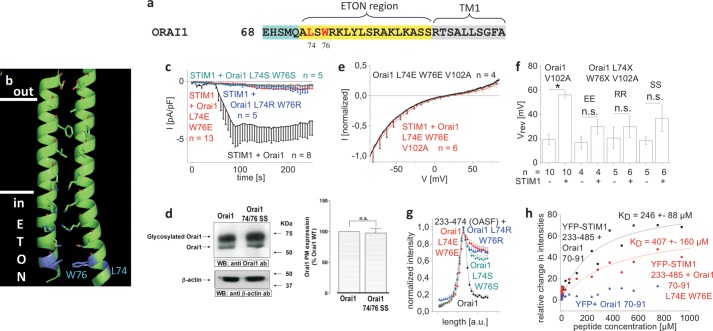
**The hydrophobic residues Leu-74 and Trp-76 of the ETON region contribute in a concerted manner to the coupling with STIM1.** *a*, amino acid sequence of Orai1 N terminus displaying Leu-74 and Trp-76 in *red* in the conserved ETON region (*yellow area*) *b*, schematic representation of two TM1 domains + ETON regions highlighting Leu-74 and Trp-76 (*blue*) in the ETON region. *c*, time course of whole cell inward currents at −74 mV activated by passive store depletion of HEK 293 cells co-expressing CFP-STIM1 and YFP-Orai1 L74S/W76S, YFP-Orai1 L74E/W76E, and YFP-Orai1 L74R/W76R in comparison with wild-type YFP-Orai1. *pF*, picofarads. *d*, Orai1 plasma membrane expression in HEK293 cells, expressing Orai1 WT in comparison with Orai1 L74S/W76S determined by biotinylation as described under “Experimental Procedures” (*n* = 5; *, *p* < 0.05). *Error bars* indicate S.E. *e*, I/V relationship of normalized Orai1 V102A currents in comparison with that of Orai1 L74E/W76E/V102A, shown as example. *Error bars* indicate S.E. *n.s.*, not significant. *WB*, Western blot; *ab*, antibody. *f*, block diagram displaying reversal potentials of Orai1 V102A, Orai1 L74S/W76S/V102A (*SS*), Orai1 L74E/W76E/V102A (*EE*), and Orai1 L74R/W76R/V102A (*RR*) in the absence and presence of STIM1 (*, *p* < 0.05). *Error bars* indicate S.E. *g*, intensity plots representing the localization of STIM1 233–474 (OASF) across the cell when co-expressed with Orai1 L74S/W76S, Orai1 L74E/W76E, and Orai1 L74R/W76R when compared with wild-type Orai1. *a. u.*, arbitrary units. *h*, binding curve fitted to a one-site binding model for YFP-STIM1 233–485 from a HEK293 cell lysate together with Orai1 NT(aa70–91) or Orai1 NT(aa70–91 L74E/W76E) in comparison with YFP from HEK293 cell lysate together with Orai1 NT(aa70–91), obtained from thermophoresis measurements.

Therefore, we focused next on double point mutations around position 76 based on our observation that Orai1 ΔN_1–74_ in contrast to Orai1 ΔN_1–76_ still retained STIM1-dependent function. Among several mutants, which were summarized in supplemental Fig. 6, we identified a double mutation of the two hydrophobic residues Leu-74 and Trp-76 that impaired channel function. Three mutants, Orai1 L74E/W76E, Orai1 L74R/W76R, and Orai1 L74S/W76S (Fig. 4, *a* and *b*), were generated by switching the hydrophobic amino acids to charged (Glu and Arg) or polar (Ser) residues. All three mutants displayed a significant reduction in STIM1-dependent activation (Fig. 4*c*), which did not result from disrupted plasma membrane targeting as evident from biotinylation experiments exemplarily shown for Orai1 L74S/W76S (Fig. 4*d*). Introduction of these mutations in the Orai1 V102A core resulted in constitutive, nonselective currents in the absence of STIM1, which displayed slight, yet nonsignificant rightward shifts of the reversal potential in the presence of STIM1 (Fig. 4, *e* and *f*). Furthermore, co-expression with OASF revealed a significant reduction in its co-localization with all three Orai1 L74E/L74R/L74S and W76E/W76R/W76S mutants (Fig. 4*g*). Accordingly, thermophoresis measurements of YFP-STIM1 233–485 and the Orai1 aa70–91 L74E/W76E N-terminal peptide revealed an about 1.7-fold increase in *K_D_* to 406 ± 68 μm when compared with the wild-type Orai1 N-terminal peptide. Nevertheless, the previously determined *K_D_* with the shorter 77–91 N-terminal peptide was at least 10-fold higher, suggesting additional residues in the ETON region between 70 and 76 that also contribute to the interaction with STIM1 (Fig. 4*h*). In summary, Leu-74 and Trp-76 were identified as hydrophobic hot spot residues within the ETON region that contribute in a concerted manner to the binding interface with STIM1.

##### The Positively Charged Arg-77 and Lys-78 in the ETON Region Also Contribute to STIM1 Binding

We focused next on the potential role of the positively charged residues in the ETON region downstream to Trp-76 as they are also frequently found in protein-protein interfaces (35). Single point mutations of positively charged residues (Orai1 R77E, Orai1 K78E, Orai1 R83A, Orai1 K87A) did not severely impair STIM1-dependent current activation when compared with wild-type Orai1 (data not shown) (25). Only the single point mutant Orai1 K85E exhibited loss of function due to impaired gating together with a reduced STIM1 binding (supplemental Fig. 7) in line with Refs. 24 and 25.

**FIGURE 5.**
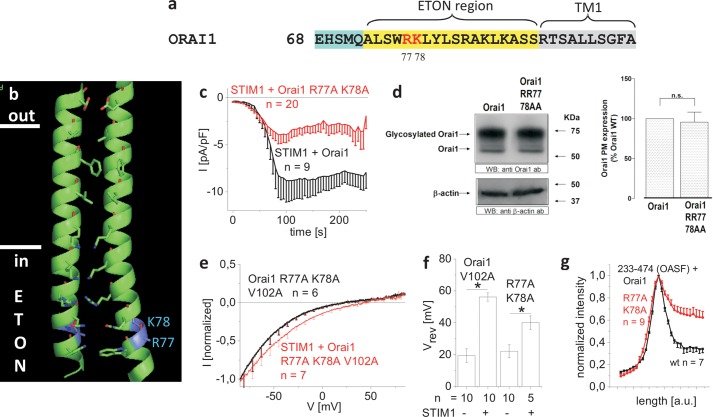
**The positively charged residues Arg-77 and Lys-78 in the ETON region also contribute to STIM1 binding.** *a*, amino acid sequence of Orai1 N terminus displaying Arg-77/Lys-78 in *red* in the ETON region (*yellow area*). *b*, schematic representation of two TM1 domains + ETON regions highlighting Arg-77 and Lys-78 (*blue*) in the ETON region. *c*, time course of whole cell inward currents at −74 mV activated by passive store depletion of HEK 293 cells co-expressing CFP-STIM1 and YFP-Orai1 R77A/K78A in comparison with wild-type YFP-Orai1. *pF*, picofarads. *d*, Orai1 plasma membrane expression in HEK293 cells, expressing Orai1 in comparison with Orai1 R77A/K78A as determined by biotinylation as described under “Experimental Procedures” (*n* = 5; *, *p* < 0.05). *Error bars* indicate S.E. *n.s.*, not significant. *WB*, Western blot; *ab*, antibody. *e*, I/V relationships of normalized Orai1 R77A/K78A/V102A currents in the absence and presence of STIM1. *Error bars* indicate S.E. *f*, block diagram displaying reversal potentials of Orai1 V102A and Orai1 R77A/K78A/V102A in the absence and presence of STIM1 (*, *p* < 0.05). *Error bars* indicate S.E. *g*, intensity plots representing the localization of STIM1 233–474 (OASF) across the cell when co-expressed with Orai1 R77A/K78A when compared with wild-type Orai1. *Error bars* indicate S.E.

Next, we constructed double point mutations and focused initially on residues Arg-77/Lys-78 adjacent to position 76 (Fig. 5, *a* and *b*). The Orai1 R77A/K78A double mutant displayed ∼50% reduced STIM1-dependent current densities (Fig. 5*c*), the attenuation of which was not caused by a diminished plasma membrane targeting (Fig. 5*d*). Introduction of this double R77A/K78A mutation into the Orai1 V102A core resulted in the expected nonselective, constitutive currents, which still exhibited a significant rightward shift of the reversal potential in the presence of STIM1 but not to the extent of the Orai1 V102A (Fig. 5, *e* and *f*). In accordance, co-localization of the Orai1 R77A/K78A mutant with OASF was partially reduced (Fig. 5*g*). Hence, we suggest that the positively charged Arg-77 and Lys-78 residues in the ETON region also contribute in a concerted manner to the STIM1 binding interface, but in a less dominant role as found for the hydrophobic Leu-74 and Trp-76.

##### The Positively Charged Arg-83 and Lys-87 Contribute to the STIM1 Binding Interface in a Concerted Manner, and as Pore-lining Residues, They Provide Gating Function to the ETON Region

**FIGURE 6.**
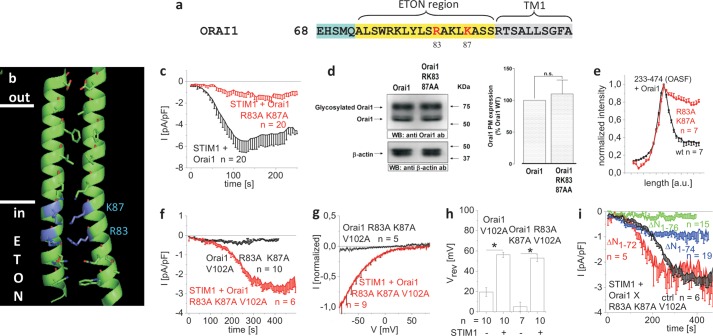
**The positively charged Arg-83 and Lys-87 residues in the ETON region contribute to both STIM1 binding and the gate.** *a*, amino acid sequence of Orai1 N terminus displaying Arg-83/Lys-87 in *red* in the ETON region (*yellow area*). *b*, schematic representation of two TM1 domains + ETON regions highlighting the positively charged residues Arg-83 and Lys-87 (*blue*) in the ETON region. *c*, time course of whole cell inward currents at −74 mV activated by passive store depletion of HEK 293 cells co-expressing CFP-STIM1 and YFP-Orai1 R83A/K87A in comparison with wild-type YFP-Orai1. *d*, plasma membrane expression in HEK293 cells expressing Orai1 WT in comparison with Orai1 R83A/K87A determined by biotinylation as described under “Experimental Procedures” (*n* = 5; *, *p* < 0.05). *Error bars* indicate S.E. *n.s.*, not significant. *WB*, Western blot; *ab*, antibody. *e*, intensity plots representing the localization of STIM1 233–474 (OASF) across the cell when co-expressed with Orai1 R83A/K87A when compared with wild-type Orai1. *Error bars* indicate S.E. *f*, time course of whole cell inward currents at −74 mV activated by passive store depletion of HEK 293 cells co-expressing CFP-STIM1 and YFP-Orai1 R83A/K87A/V102A in the absence and presence of STIM1. *g*, I/V relationships of normalized Orai1 R83A/K87A/V102A currents in the absence and presence of STIM1. *h*, block diagram displaying reversal potentials of Orai1 V102A and Orai1 R83A/K87A/V102A in the absence and presence of STIM1 (*, *p* < 0.05). *Error bars* indicate S.E. *i*, time course of whole cell inward currents at −74 mV activated by passive store depletion of HEK 293 cells co-expressing CFP-STIM1 and YFP-Orai1 ΔN_1–72_ R83A/K87A/V102A, YFP-Orai1 ΔN_1–74_ R83A/K87A/V102A, and YFP-Orai1 ΔN_1–76_ R83A/K87A/V102A in comparison with YFP-Orai1 R83A/K87A/V102A (*ctrl*).

Next, double point mutation of the pore-lining positively charged residues Arg-83 and Lys-87 (Fig. 6, *a* and *b*) to alanines (Orai1 R83A/K87A) resulted in a substantial reduction of STIM1-dependent current activation (Fig. 6*c*). The reduced current density of Orai1 R83A/K87A was not caused by an impaired plasma membrane targeting as determined by biotinylation experiments (Fig. 6*d*). Co-localization with OASF was, however, reduced as evident from density profiles (Fig. 6*e*), suggesting a partially reduced interaction with STIM1. Strikingly, the introduction of these mutations into the Orai1 V102A core yielded a channel with novel characteristics (Fig. 6, *f–h*) in that the Orai1 R83A/K87A/V102A mutant displayed complete loss of function (Fig. 6, *f* and *h*). Hence, the concerted alanine substitutions at positions 83 and 87 apparently altered the geometry of the elongated pore in such a way that Ca^2+^ permeation was inhibited. In support of this, 2-aminoethoxydiphenyl borate failed to evoke currents from the double Orai1 R83A/K87A mutant compatible with a severe defect in the pore (supplemental Fig. 3*g*). It is tempting to speculate that the positive charges of the pore-lining residues Arg-83 and Lys-87 in the ETON region (15) contribute to the stabilization of the elongated pore, the alanine mutation of which leads to pore collapse.

An overall disturbance of channel function could be excluded as Orai1 R83A/K87A/V102A was still activated by STIM1, leading to inward-rectifying Ca^2+^ currents that showed a rightward shift of the reversal potential similar to STIM1+Orai1 V102A (Fig. 6, *f–h*). Thus, interaction with STIM1 was still attainable with the R83A/K87A mutations in the ETON region, although the STIM1-dependent currents activated at a slower rate and to a lower extent than those obtained with Orai1 V102A.

As the activity of the Orai1 R83A/K87A/V102A mutant was strictly STIM1-dependent, we utilized it to confirm the impact of N-terminal truncations presented in Fig. 1. Consistently, Orai1 ΔN_1–72_ R83A/K87A/V102A and Orai1 ΔN_1–74_ R83A/K87A/V102A regained function in the presence of STIM1, whereas Orai1 ΔN_1–76_ R83A/K87A/V102A failed to activate, independent of the presence or absence of STIM1 (Fig. 6*i*). In summary, our results suggest an additional role of the pore-lining positively charged Arg-83 and Lys-87 residues in providing a gating function to the ETON region that, in conjunction as a binding interface for STIM1, fine-tunes pore width for controlled STIM1-dependent Ca^2+^ entry.

## DISCUSSION

The gating of Orai1 channels requires both a robust STIM1 coupling to the Orai1 C terminus and a weaker one to the Orai1 N terminus, respectively (12, 22). The Orai1 N-terminal interaction is mediated via the conserved ETON region as demonstrated by systematic N-truncations as well as point mutations. Our study revealed multiple hot spots along the ETON region that contribute in a concerted manner to the STIM1 binding interface. In particular, the hydrophobic residues Leu-74 and Trp-76 clustered at the beginning of the ETON region are molecular determinants in the coupling to STIM1, the mutation of which decreased affinity to and almost abolished Orai1 activation via STIM1. The positively charged pore-lining residues Arg-83 and Lys-87 additionally function as a gate that likely shapes the exit at the cytosolic side by electrostatic tuning of the width and barrier of the elongated pore. Hence, the ETON region combines both binding interface and gate for the activation of Orai1 via STIM1, in line with the recently published crystal structure (15).

The relevance of the conserved ETON region (aa73–90) in triggering STIM1-mediated Orai1 activation has already been reported by several studies (22, 31, 33, 36), in that N-terminal truncations up to aa 74 maintain a functional channel, whereas a deletion up to aa 80 (33) or of the whole N terminus of Orai1 abolishes STIM1-dependent activation. Consistently, deletion of aa 74–85 (24) leads to nonfunctional Orai1 channels. In this study, we initially focused on characterizing the minimal domain of the ETON region that is essential for retaining STIM1-dependent activation of Orai1 currents. The minimal stretch, based on progressive N-terminal Orai1 truncations, included residues 76–90 of the ETON region, which still allowed at least partial STIM1-dependent activation. Residues upstream of 76, *i.e.* 72–75, also contributed to STIM1 binding as their deletion progressively reduced current densities, but not as pronounced as with deletion of the first 75 amino acids. Additional deletion of residue Trp-76 completely abolished STIM1-dependent Orai1 function. Park *et al.* (22) have reported weak binding of the CAD to the Orai1 N-terminal fragments aa1–91 and aa48–91 but not to Orai1 1–70 in support of STIM1 interaction with the conserved ETON region. Consistent with the proposed weak interaction of the ETON region, a peptide fragment aa70–91 and STIM1 C-terminal fragment interacted with a *K_D_* of ∼250 μm as determined by thermophoresis experiments compatible with a weak, transient interaction (37, 38). The *K_D_* shifted drastically above 4 mm with a shortened ETON region comprising aa77–91. Hence, this result, in line with the N-truncation Orai1 mutants, suggests Trp-76 together with residues 72–75 as a hot spot within the binding interface of the ETON region for interaction with STIM1. Thus, STIM1 requires almost the whole ETON region for STIM1-dependent Orai1 activation.

To further pinpoint hot spot residues mediating STIM1 binding, we carried out scanning mutagenesis of selected hydrophobic as well as positively charged residues in the ETON region as those amino acids are frequently found in protein-protein interfaces (35). Single point mutations of Trp-76 (except of a proline) within the full-length Orai1 failed to drastically impair STIM1-dependent Orai1 function. However, the double mutation of Leu-74 together with Trp-76 to more polar residues substantially reduced Orai1 activation consistent with a significant decrease in co-localization and affinity of STIM1 C-terminal fragments. Thus, Leu-74 and Trp-76 represent hydrophobic hot spot residues within the ETON region contributing in a concerted manner to the Orai1 N-terminal coupling with STIM1. The single W76P substitution likely introduced a break in the ETON helical region that severely interfered with the overall orientation of the STIM1 binding interface.

In addition to these hydrophobic residues, we further characterized positively charged residues along the ETON region that contributed to the interaction with STIM1. Except for the single amino acid mutant Orai1 K85E (24, 25), which has already been reported to abolish Orai1 channel function, we identified double mutations of positively charged residues, *i.e.* Arg77/Lys-78 and Arg-83/Lys-87, along the ETON region that diminished both current activation and STIM1 co-localization. Although Arg-77 and Lys-78 are located on the pore-averted side of the helical ETON region, Arg-83 and Lys-87 represent pore-lining residues close to the membrane as derived from the recent Orai crystal structure (15). Therefore, the latter two residues together with Arg-91 have been recently hypothesized to form a potential gate of the Orai1 channel (16). Utilization of the constitutively active Orai1 V102A mutant indeed enabled us to reveal a gating function of Arg-83 and Lys-87 as their mutation to alanines abolished Orai1 Arg-83/Lys-87 V102A channel function that was restored by the presence of STIM1. We (this work) and others (24) employed the Orai1 V102A mutant as an alternative and sensitive tool to monitor N-terminal interactions with STIM1 as monitored by restored Ca^2+^ selectivity as long as STIM1 coupling occurred. The striking loss of function of the generally constitutively active Orai1 V102A mutant upon alanine mutations of Arg-83 and Lys-87 suggested these residues as gating elements that provide both electrostatic stabilization and barrier to the pore jointly formed by the ETON regions in the closed conformation of the Orai1 channel complex. The presence of STIM1 restored function of this R83A/K87A Orai1 V102A mutant probably by tuning the geometry of the pore for Ca^2+^ permeation. In accordance, charge swap in the R83E/K87E Orai1 V102A mutant resulted in a constitutively active channel, supporting the concept of electrostatic pore stabilization (supplemental Fig. 8).

In summary, almost the whole ETON region functions as binding interface for Orai1 interaction with STIM1 and additionally provides electrostatic gating elements to fine-tune the shape of the elongated pore. STIM1 binding onto the ETON region occurs with a weak affinity in the μm range (37, 38) and requires at least the N-terminal strand starting with residue 76. The residues Leu-74 and Trp-76 acting in a concerted manner represent a hydrophobic hot spot interface for the Orai1 N-terminal interaction with STIM1 that is additionally affected by positively charged residues within the ETON region. Besides Lys-85 (24, 25), we identified the two pore-lining residues Arg-83 and Lys-87 that exert a gating function both by electrostatic stabilization and by barrier to the elongated pore of the closed channel. The identification of the hot spot residues may stimulate the search for small molecule Orai1 inhibitors enabling targeted disruption of STIM1 interaction with the ETON region.

## Footnotes

4 The abbreviations used are:

CRAC: calcium release-activated calcium
STIM1: stromal interaction molecule 1
ETON: extended transmembrane Orai1 N-terminal
aa: amino acid(s)
NT: N terminus
TM1: transmembrane helix 1.
